# Birth order, family size, and the risk of cancer in young and middle-aged adults

**DOI:** 10.1054/bjoc.2001.1811

**Published:** 2001-06

**Authors:** K Hemminki, P Mutanen

**Affiliations:** Department of Biosciences at Novum, Karolinska Institute On leave from the Finnish Institute of Occupational Health, 141 57 Huddinge, Helsinki, Finland, Sweden

**Keywords:** cancer risk, breast cancer, melanoma, perinatal factors, anthropomorphic factors

## Abstract

We used the Swedish Family-Cancer Database to analyse the effects of birth order and family size on the risk of common cancers among offspring born over the period 1958–96. Some 1.38 million offspring up to age 55 years with 50.6 million person-years were included. Poisson regression analysis included age at diagnosis, birth cohort, socio-economic status and region of residence as other explanatory variables. The only significant associations were an increasing risk for breast cancer by birth order and a decreasing risk for melanoma by birth order and, particularly, by family size. When details of the women's own reproductive history were included in analysis, birth orders 5–17 showed a relative risk of 1.41. The effects on breast cancer may be mediated through increasing birth weight by birth order. For melanoma, socio-economic factors may be involved, such as limited affordability of sun tourism in large families. Testis cancer showed no significant effect and prostate cancer was excluded from analysis because of the small number of cases. © 2001 Cancer Research Campaign http://www.bjcancer.com


British Journal of Cancer (2001) 84(11), 1466–1471
© 2001 Cancer Research Campaign
doi: 10.1054/ bjoc.2001.1811, available online at http://www.idealibrary.com on IDEAL®

### Birth order, family size, and the risk of cancer in young and middle-aged adults

**K Hemminki and P Mutanen[1]**

Department of Biosciences at Novum, Karolinska Institute, 141 57 Huddinge, Sweden; [1]On leave from the Finnish Institute of Occupational Health, Helsinki, Finland

**Summary** We used the Swedish Family-Cancer Database to analyse the effects of birth order and family size on the risk of common cancers among offspring born over the period 1958–96. Some 1.38 million offspring up to age 55 years with 50.6 million person-years were included. Poisson regression analysis included age at diagnosis, birth cohort, socio-economic status and region of residence as other explanatory variables. The only significant associations were an increasing risk for breast cancer by birth order and a decreasing risk for melanoma by birth order and, particularly, by family size. When details of the women's own reproductive history were included in analysis, birth orders 5–17 showed a relative risk of 1.41. The effects on breast cancer may be mediated through increasing birth weight by birth order. For melanoma, socio-economic factors may be involved, such as limited affordability of sun tourism in large families. Testis cancer showed no significant effect and prostate cancer was excluded from analysis because of the small number of cases. © 2001 Cancer Research Campaign http://www.bjcancer.com

**Keywords**: cancer risk; breast cancer; melanoma; perinatal factors; anthropomorphic factors

Birth order and family size may affect cancer risk among offspring in several ways. As genetic risk factors, early-onset cancers or other inherited diseases may limit the reproductive period of the parents and show higher risks for small families because of selection (Hemminki and Kyyrönen, 1999). Several possible biological risk factors can be identified. Birth weight increases with maternal parity, apparently due to growth-promoting effects, such as increased estrogen levels, during the intrauterine period (Andersson et al, 2000; Ekbom et al, 1997; Juntunen et al, 1997; Kaijser et al, 2000; Trichopoulos, 1990). Birth weight is a risk factor for breast cancer, and some, but not all studies have found a correlation between birth order and risk of breast cancer (Hsieh et al, 1991; Janerich et al, 1994; Michels et al, 1996; Potischman and Trosi, 1999; Sanderson et al, 1996). The question of birth order has also been investigated in testicular, prostate and childhood cancers, showing protective effect of high birth order for testicular cancer but no uniform effects for the other malignancies (Emerson et al, 1991; Hsieh et al, 1999; Kaye et al, 1991; Moller and Skakkebaek, 1997; Sabroe and Olsen, 1998; Shaw et al, 1984; Shu et al, 1988; Westergaard et al, 1998). High birth order often correlates with high parental age at conception, but has not been found to be an important risk factor for cancer in offspring (Colditz et al, 1991; Hemminki and Kyyrönen, 1999; Hemminki et al, 1999; Janerich et al, 1994). Large families involve intimate contacts between the family members, with potential consequences in terms of infectious diseases. Thus, family size correlates with the probability of infection with *Helicobacter pylori*, a gastric cancer pathogen. Various socio-economic and cultural factors are relevant to large families. In contrast to the potential impact of the birth order and family size parameters to risk of cancer, relatively few studies have explored them.

We used the nationwide Swedish Family-Cancer Database to assess the effects of birth order and family size on the subsequent risk of cancer. In order to rule out effects of familial and inherited cancers, offspring were included only from families in which both parents were cancer-free. Poisson regression models were used together with several possible intervening variables. The study included 1.38 million offspring accumulating 50.6 million person-years during the follow-up period from 1958 to 1996.

*Received 27 November 2000*
*Revised 22 February 2001*
*Accepted 6 March 2001*

*Correspondence to:* K Hemminki

### SUBJECTS AND METHODS

### Registers and source of subjects

The Family-Cancer Database was formed by linkage between the Second-Generation Register, the Swedish Cancer Registry, the National Census of 1960 and the Death Notification Registry (Hemminki and Vaittinen, 1998). In the Second-Generation Register, maintained by Statistics Sweden, children born in Sweden from 1935 to 1996 were registered with their biologic parents as families.

A four-digit diagnostic code adopted from the 7th revision of the *International Classification of Diseases* was used. Cancer site groupings are shown in Tables 1 and 2. Special groupings were: oral (140–141.9, 143.0–148.9, 161.0–161.9), rectum excluding anus (154–154.0, 154.8), liver and gall bladder (155.0–156.9), lung (162.0–163), other genital organs (154.1, 176.0–176.9, 179.0 –179.9), endometrium (172.0–172.9, 174.0–174.4), lymphoma (200.0–202.9), leukaemia and myelofibrosis (204.0–209.9). The first primary cancers were diagnosed in offspring during years 1958–1996 but sites were included in our analysis only if at least 100 cases were found for either sex.

**Table 1** Poisson regression analysis of male cancer. RR and 95% confidence limits for birth order and family size, adjusted for SES 1960, region 1960, age and birth cohort

| Cancer type | | | Birth order | | | Family size | | |
|---|---|---|---|---|---|---|---|---|
| ICD-7 | | Cancer cases (*n*) | RR and 95% CI | | | RR and 95% CI | | |
| 140+ | Oral | 281 | 0.95 | 0.80 | 1.12 | 1.08 | 0.98 | 1.19 |
| 151 | Stomach | 151 | 1.04 | 0.83 | 1.31 | 0.92 | 0.79 | 1.06 |
| 153 | Colon | 391 | 1.09 | 0.95 | 1.25 | **0.92** | **0.84** | **1.00** |
| 154-a | Rectum,- anus | 210 | 0.94 | 0.78 | 1.14 | 1.05 | 0.94 | 1.18 |
| 179+a | Other genitals, anus | 73 | 1.15 | 0.88 | 1.49 | 1.15 | 0.95 | 1.38 |
| 155-6 | Liver, gall bladder | 95 | 0.97 | 0.74 | 1.27 | 1.09 | 0.92 | 1.29 |
| 157 | Pancreas[1] | 100 | 0.99 | 0.68 | 1.45 | 0.90 | 0.71 | 1.16 |
| 162–3 | Lung | 390 | 1.11 | 0.97 | 1.28 | 1.00 | 0.92 | 1.09 |
| 178 | Testis | 1289 | 0.94 | 0.87 | 1.01 | 0.97 | 0.92 | 1.02 |
| 180 | Kidney | 260 | 0.89 | 0.74 | 1.08 | 1.02 | 0.92 | 1.13 |
| 181 | Urinary bladder | 366 | 0.93 | 0.81 | 1.08 | **1.10** | **1.01** | **1.20** |
| 190 | Melanoma, skin | 1101 | 0.96 | 0.89 | 1.05 | 0.95 | 0.90 | 1.01 |
| 191 | Skin, SCC | 210 | 1.00 | 0.83 | 1.20 | 1.00 | 0.88 | 1.12 |
| 193 | Brain, nervous system | 1328 | **0.93** | **0.86** | **0.99** | 1.02 | 0.98 | 1.07 |
| 194 | Thyroid | 166 | 0.85 | 0.69 | 1.04 | 1.12 | 0.99 | 1.27 |
| 195 | Endocrine, other | 393 | 0.90 | 0.78 | 1.03 | 1.01 | 0.92 | 1.10 |
| 196 | Bone | 244 | 0.88 | 0.76 | 1.03 | 1.10 | 0.99 | 1.22 |
| 197 | Connective tissues | 179 | 1.10 | 0.91 | 1.32 | 0.95 | 0.83 | 1.09 |
| 200–2 | Lymphoma | 1157 | 1.00 | 0.93 | 1.08 | 0.96 | 0.91 | 1.01 |
| 204–9 | Leukaemia | 760 | 1.01 | 0.93 | 1.11 | 1.00 | 0.94 | 1.07 |
| Other | Other cancers | 299 | 0.99 | 0.85 | 1.16 | 0.99 | 0.90 | 1.10 |
| All | All cancers | 9995 | **0.97** | **0.95** | **1.00** | 0.99 | 0.98 | 1.01 |

[1] Pancreas: only family size and birth order 1,2,3–4 included in analysis. Significant values are in bold.

The main explanatory variables were birth order and family size. Family size indicated the number of offspring in the family (grouped 1, 2, 3–4, 5–17). It is assumed that family size is same for all children in one family and is the same during follow-up time for the children. Family size can also by seen as parity for the mother of the family. Birth order expressed the order of birth of the child into the family (grouped 1, 2, 3–4, 5–17). The parents of a child were recorded at his or her birth. In case of divorce, we had no possibility of verifying which children remained in the same family. However, usually all children have remained with the mother. Other explanatory variables included in statistical analysis were socio-economic status (SES, 4-category variable: agriculture, professional, worker, other) and area of living (region, 5-category variable: Stockholm area, the largest city; Göteborg-Malmö area, 2 largest cities in south of Sweden; Götaland, Svealand and Norrland, 3 geographic regions, from south to north, respectively). Details of birth order and family size were extracted from the Census 1960 of Statistics Sweden. All analyses also included two other variables: age at diagnosis (5-year categories in the range from 0 to 61, last age group being 55–61) and year of birth (birth cohort, three categories: 1941–1950, 1951–1960; 1961–1996). The calendar period of follow-up (four 10-year categories: 1958–1967; 1968–1977; 1978–1987; 1988–1996) was included in breast cancer analysis instead of year of birth. Two further explanatory variables were included for female breast cancer offspring's own parity (categories: 0, 1, 2, 3, 4+) and the age at which she bore her own first child (categories: no child; 10–19; 20–24; 25–29; 30–34; 35+). The offspring's own parity variable was determined by information available at the end of study period (i.e. 1996), and is subject to truncation due to termination of follow-up or, in rare instances, diagnosis of early-onset cancer.

Our study population consisted of subjects in the Family-Cancer Database whose mother's first child was born during 1941–1960. This criterion was used to include complete sibships. Both parents had to have a valid known Swedish personal identification number. The child was included only when both of his or her parents had no cancer observed until the end of follow-up period (i.e. 1996). Also excluded were those children for whom the socio-economic status or area of living were unknown.

The study population consisted of 1.38 million offspring (711 203 males and 670 840 females). The follow-up of this population during 1958–1996 gave a total of 50.6 million person-years (26.0 million and 24.6 million person-years for males and females, respectively). The mean follow-up times were 35.8 and 35.9 years for males and females respectively.

### Statistical methods

The follow-up period was 1958–1996 for first primary cancers. Every offspring in the study population was followed from the birth or from the beginning year of the follow-up period (= 1958). Follow-up ended when the offspring presented with cancer, died, moved out of the country or at the end of follow-up period (= 1996), whichever came first.

Person-years and cancer cases were counted and grouped by the study explanatory variables (family size, birth order, SES and region) during the follow-up period for the child. The Poisson regression method (multiplicative model and logarithm of person years as offset) was applied to the data and the GENMOD-procedure of the SAS-system was used. Very small cells in the data set (<50 person years) were excluded at the analysis stage. The term rate ratio (RR) was used for the exp(b), where b is the estimated model parameter value; this was interpreted as incidence rate ratio (e.g. RR is the incidence rate ratio for the birth order category 2 as compared to birth order category 1 as the reference category).

*© 2001 Cancer Research Campaign* 

**Table 2** Poisson regression analysis of female cancer. RR and 95% confidence limits for birth order and family size, adjusted for SES 1960, region 1960, age and birth cohort

| ICD-7 | Cancer type | Cancer cases (n) | Birth order RR and 95% CI | | | Family size RR and 95% CI | | |
|---|---|---|---|---|---|---|---|---|
| 140+ | Oral | 148 | 0.97 | 0.78 | 1.21 | 1.04 | 0.91 | 1.19 |
| 151 | Stomach | 156 | 1.14 | 0.93 | 1.41 | 0.96 | 0.84 | 1.10 |
| 153 | Colon | 483 | 1.02 | 0.91 | 1.15 | 0.96 | 0.89 | 1.04 |
| 154-a | Rectum,- anus | 169 | 0.94 | 0.75 | 1.18 | 0.99 | 0.87 | 1.13 |
| 176+a | Other genitals, anus | 128 | 1.13 | 0.91 | 1.41 | 1.02 | 0.88 | 1.18 |
| 155–6 | Liver, gall bladder | 111 | 1.19 | 0.91 | 1.55 | 0.89 | 0.75 | 1.06 |
| 157 | Pancreas [1] | 76 | 0.79 | 0.51 | 1.23 | 0.92 | 0.69 | 1.22 |
| 162–3 | Lung | 359 | **1.18** | **1.02** | **1.37** | 0.97 | 0.89 | 1.06 |
| 170 | Breast | 4819 | **1.05** | **1.01** | **1.10** | **0.96** | **0.94** | **0.99** |
| 171 | Cervix | 1655 | 1.04 | 0.98 | 1.11 | 1.01 | 0.96 | 1.05 |
| 172/4 | Endometrium | 397 | 1.01 | 0.86 | 1.18 | 0.95 | 0.88 | 1.04 |
| 175 | Ovary | 937 | 1.03 | 0.95 | 1.13 | 0.99 | 0.94 | 1.05 |
| 180 | Kidney | 151 | 1.02 | 0.81 | 1.28 | 0.99 | 0.87 | 1.14 |
| 181 | Urinary bladder | 125 | 1.22 | 0.95 | 1.57 | **0.83** | **0.70** | **0.99** |
| 190 | Melanoma, skin | 1631 | 0.95 | 0.88 | 1.02 | **0.91** | **0.87** | **0.95** |
| 191 | Skin, other | 144 | 1.03 | 0.83 | 1.28 | 1.04 | 0.90 | 1.19 |
| 193 | Brain, nervous system | 1291 | 1.03 | 0.96 | 1.10 | 0.99 | 0.94 | 1.04 |
| 194 | Thyroid, gland | 598 | **0.89** | **0.80** | **0.99** | 1.00 | 0.93 | 1.07 |
| 195 | Endocrine, other | 556 | 0.99 | 0.88 | 1.10 | 1.01 | 0.93 | 1.08 |
| 196 | Bone | 147 | 0.98 | 0.79 | 1.21 | 0.96 | 0.82 | 1.11 |
| 197 | Connective tissues | 195 | **0.82** | **0.67** | **1.00** | 0.99 | 0.88 | 1.12 |
| 200–2 | Lymphoma | 639 | 0.97 | 0.88 | 1.08 | 1.00 | 0.94 | 1.07 |
| 204–9 | Leukaemia | 569 | 0.91 | 0.82 | 1.01 | 1.07 | 0.99 | 1.15 |
| Other | Other cancers | 280 | 1.00 | 0.85 | 1.18 | 0.93 | 0.84 | 1.03 |
| All | All cancers | 16147 | 1.01 | 0.99 | 1.03 | **0.97** | **0.96** | **0.99** |

[1]Pancreas: only family size and birth order 1,2,3–4 included in analysis. Significant values are in bold.

### RESULTS

Table 1 shows the effects of birth order and family size on cancers in males in the Poisson regression analysis, adjusting for age, birth cohort, SES and region. The risk for nervous system and all cancer decreased with birth order, but the effect was only marginally significant. The family size variable was associated with a decreasing risk for colon cancer and melanoma, and an increased risk for bladder cancer, though again all with borderline significance. The effects of family size at four selected sites, colon, lung, bladder and skin (melanoma) are shown in Figure 1. For colon, lung and bladder the trend was not consistent and none of the RRs in large families (5–17 children) deviated significantly from those of one-child families. For melanoma the data were more consistent, and the RR in large families was 0.7.

Among women, birth order was associated with an increased the risk for lung and breast cancer, and a decreased risk for thyroid and connective tissue cancer (Table 2). Family size was associated with an opposite effect for breast cancer, and it also decreased the risk for bladder cancer and melanoma. When the effects of birth order were examined graphically, only breast cancer showed a systematic trend, higher birth order increasing the risk (Figure 2). The RRs for breast were 1.03, 1.12 and 1.41 for the birth order groups 2, 3–4 and 5–17, respectively. For family size, the effect was consistent both for breast cancer and melanoma, risks being smaller for large families (Figure 3). For melanoma, family size exerted a larger effect than birth order. The absolute change in RR for breast cancer was modest, though significant. Thus, the direction of risk between birth order and family size was uniform for melanoma but opposite for breast cancer.

No graphical data are shown for the other significant sites in Tables 1 and 2 because the data were not systematic between categories nor were they consistent between genders.

### Further analysis of female breast cancer

Two explanatory variables were included in further analysis of breast cancer in females. These were women's own parity and age at their first childbirth. The age at diagnosis of breast cancer was restricted to 25–61 years (the oldest age group considered being 50–61 years). The modelling results are presented in Table 3. Model M1 was the starting point in the analysis, corresponding to the result in Table 2. Inclusion of the woman's own parity and her age at the first childbirth (Models 2 and 3) did not change the effect of birth order but reduced the effect of family size. The

**Table 3** Poisson regression analysis for breast cancer, including women's own parity and age at first childbirth in the model. RR and 95% confidence limits for birth order and family size. Birth order, family size and age variable are treated as continuous variables, other variables are nominal

| Model | Cancer cases (*n*) | Birth order RR and 95% CI | | | Family size RR and 95% CI | | |
|---|---|---|---|---|---|---|---|
| M1 | 4810 | 1.05* | 1.01 | 1.09 | 0.96** | 0.94 | 0.98 |
| M2 | 4586 | 1.05* | 1.01 | 1.10 | 0.98 NS | 0.95 | 1.01 |
| M3 | 4586 | 1.05* | 1.01 | 1.10 | 0.98 NS | 0.95 | 1.01 |

*$P$<0.05, **$P$<0.01, NS non-significant. M1: Birth order, family size, SES 1960, region 1960, age, birth cohort. M2: M1+ parity and age at first childbirth. M3: M1+ parity, age at first childbirth and interactions (age*parity, age* age at Ist child).

*© 2001 Cancer Research Campaign*

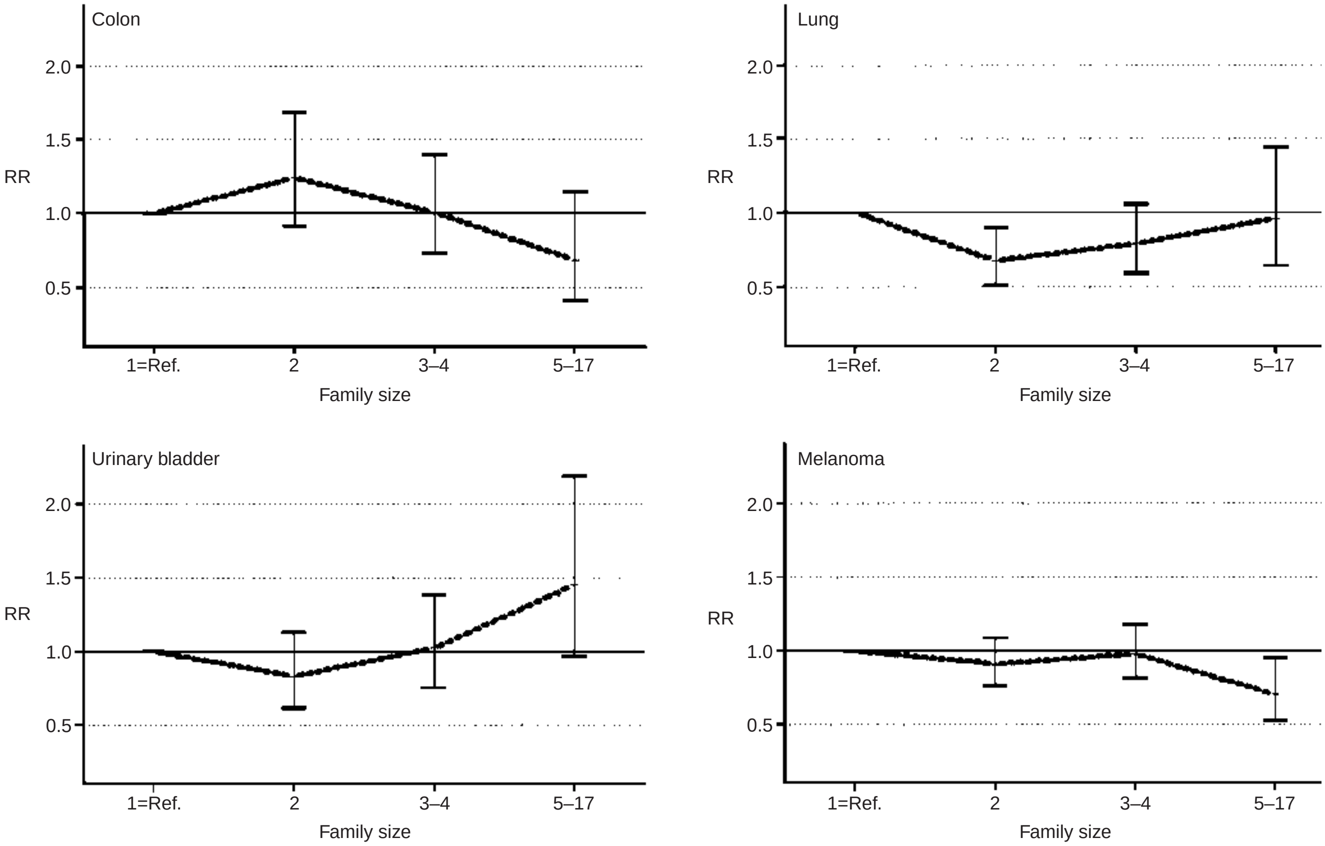

**Figure 1** Rate ratios (RR) for colon (153), lung (162–3), urinary bladder (181) and melanoma (190) in men by family size. Adjusted for age at diagnosis, birth cohort, birth order, SES and region

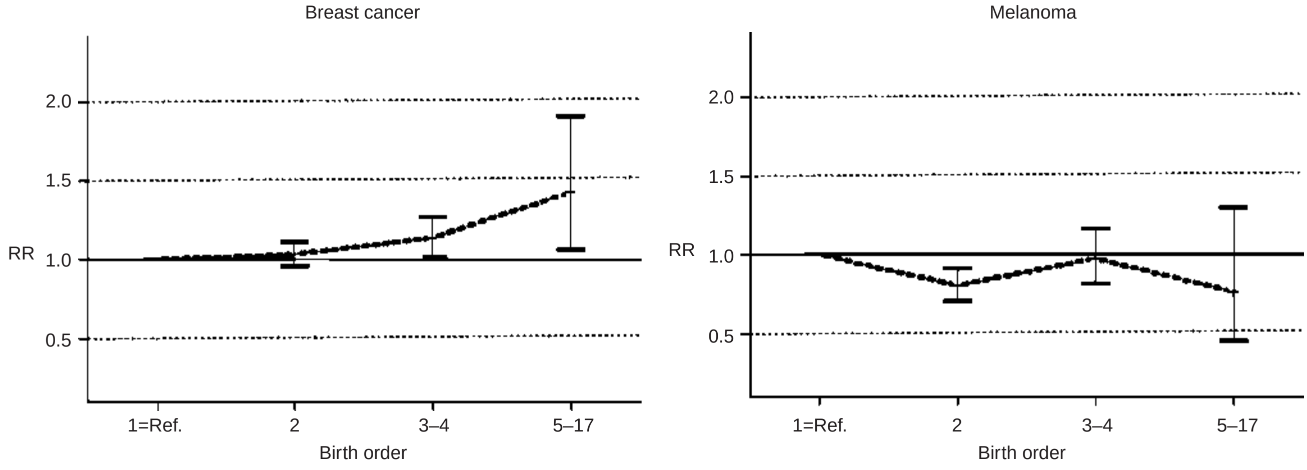

**Figure 2** Rate ratios (RR) for breast cancer (170) and melanoma (190) in women by birth order. Adjusted for age at diagnosis, birth cohort, birth order, SES and region outcome of Model 3 is shown in Figure 4. It was found that women from large families tended to have small own families, low parity but not nulliparity, and consequently a low risk of breast cancer.

### DISCUSSION

Anthropometric variables, such as birth order and family size could potentially have large population effects on cancer, because they affect every individual. As mentioned earlier, a number of plausible mechanisms have been offered to explain such effects. The conclusion from the present study is that excluding two cancer sites, birth order and family size have no major effect on the risk for the common cancers that were studied. There are three qualifications. First, the population of offspring studied was relatively young, namely those born after 1940 (mother's first child born between 1941 and 1960), so the greatest age was only 55 years (except in the case of breast cancer mentioned above). The Family-Cancer

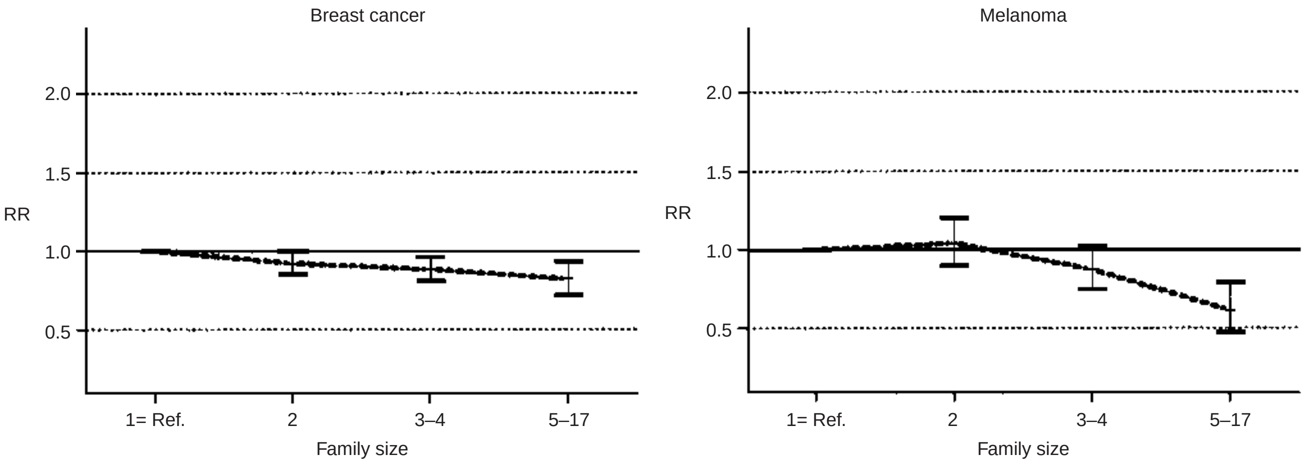

**Figure 3** Rate ratios (RR) for breast cancer (170) and melanoma (190) in women by family size. Adjusted for age at diagnosis, birth cohort, birth order, SES and region

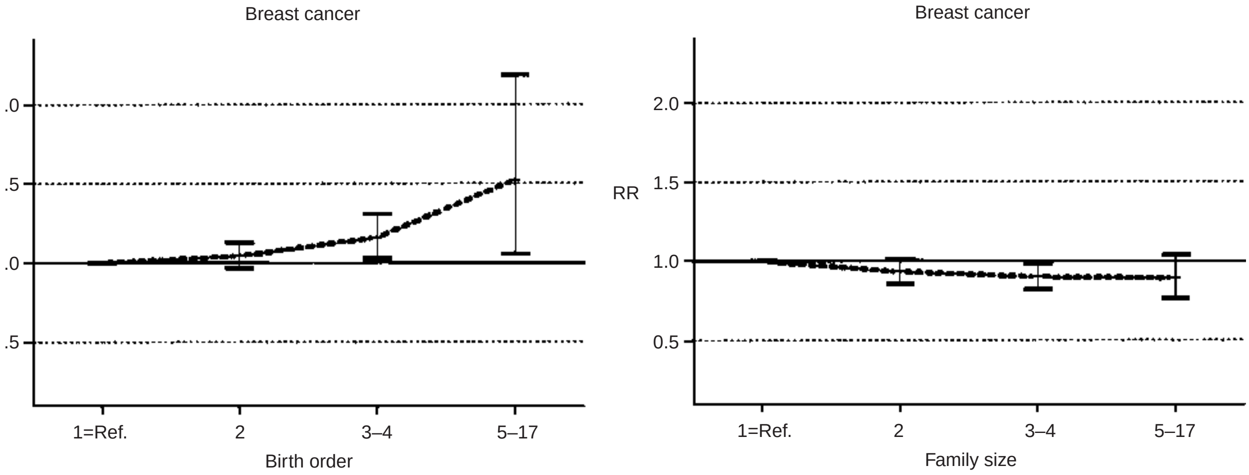

**Figure 4** Rate ratios (RR) for breast cancer (170) in females by birth order and family size (model M3). Adjusted variables in model M3 are: age at diagnosis, calendar period, birth order, family size, socio-economic status (SES), living area (county), own parity and age at first childbirth

Database includes offspring born since 1935 but because we wanted complete families, it was necessary to limit the birth years on both ends of the available birth years. Because of the age limit and the minimal eligibility requirement of 100 recorded cancers, some sites, such as prostate, were excluded from the study. Second, families were selected in which neither parent was diagnosed with cancer during the follow-up period. Thus, by definition, only sporadic cancers were included. Early-onset familial cancers may interfere with family planning and cause undefined selections leading to bias. A previous study on parental age effects from this database identified some of the problems relating to familial cancers (Hemminki and Kyyrönen, 1999). Third, the results are not informative for certain sites; thus, only strong effects would be observed among childhood brain cancers and leukaemias because they represent a small proportion of cases considered.

The two cancers in which effects noted were breast cancer and melanoma. For melanoma both birth order and, particularly, family size showed a protective effect. We cannot offer any other explanation than in large families the affordability of sun holidays in southern countries is less than in small families. The stronger effect of family size than birth order is consistent with the interpretation. Our crude socio-economic adjustment was probably not enough to account for this effect. Thus sunburns in childhood and adolescence were suggested as the explanation (English et al, 1997). For breast cancer, the data are consistent with well-documented increasing birth weights at consecutive child births, and the correlation of birth weight and breast cancer risk (Andersson et al, 2000; Juntunen et al, 1997; Kaijser et al, 2000; Michels et al, 1996; Sanderson et al, 1996; Trichopoulos, 1990). Our results agree with the main literature but not with two case-control studies that were smaller than the present study (Hsieh et al, 1991; Janerich et al, 1994; Potischman and Trosi, 1999).

Our findings on testicular cancer differ somewhat from much previous work and we found no significant protection (RR 0.94, 95 %CI 0.87–1.01) by high birth order (Moller and Skakkebaek, 1997; Sabroe and Olsen, 1998; Westergaard et al, 1998; and the

© 2001 Cancer Research Campaign cited references). Most of these studies have been well conducted case-control studies; though the most recent cohort study from Denmark only included half as many cases as the present study (Westergaard et al, 1998). Our Poisson regression analysis of testicular cancer showed strong effects of birth cohort, region and even socio-economic status. As the incidence of testicular cancer has been increasing in the countries where most of the studies have been carried out, including Sweden, the control of intervening variables may be problematic.

Taken together, our results suggest that the birth order and family size variables may be relevant only to breast cancer and melanoma among common cancer sites. For breast cancer, a plausible biological mechanism exists, and the birth order effect should be considered in designing epidemiological studies. For melanoma, socio-economic factors may underlie the findings but they may be limited to regions and countries where solar tourism is common.

### ACKNOWLEDGEMENTS

The study was supported by the Cancer Fund and the King Gustaf V Jubilee Fund.

### REFERENCES

Andersson S, Niklasson A, Lapidus L, Hallberg L, Bengtsson C and Hulten L (2000) Sociodemographic characteristics influencing birth outcome in Sweden, 1908–1930. Birth variables in The Population Study of Women in Gothenburg. *J Epidemiol Community Health* **54**: 26–78

Blaser M, Chyou P and Nomura A (1995) Age at establishment of *Helicobacter pylori* infection and gastric carcinoma, gastric ulcer, and duodenal ulcer risk. *Cancer Res* **55**: 562–565

Colditz GA, Willett WC, Stampfer MJ, Hennekens CH, Rosner B and Speizer FE (1991) Parental age at birth and risk of breast cancer in daughters: a prospective study among US women. *Cancer Causes Control* **2**: 31–36

Ekbom A, Hsieh C-C, Lipworth L, Adami H-O and Trichopoulos D (1997) Intrauterine environment and breast cancer risk in women: a population-based study. *J Natl Cancer Inst* **89**: 71–76

Emerson J, Malone K, Darling J and Starzyk P (1991) Childhood brain tumor risk in relation to birth characteristics. *J Clin Epidemiol* **44**: 1159–1166

English D, Armstrong B, Kricker A and Fleming C (1997) Sunlight and cancer. *Cancer Causes Cotrol* **8**: 271–283

Goodman K and Correa P (2000) Transmission of *Helicobacter pylori* among siblings. *Lancet* **355**: 358–362

Hemminki K and Vaittinen P (1998) National database of familial cancer in Sweden. *Genet Epidemiol* **15**: 225–236

Hemminki K and Kyyrönen P (1999) Parental age and risk of sporadic and familial cancer in offspring: implications for germ cell mutagenesis. *Epidemiology* **10**: 747–751

Hemminki K, Kyyrönen P and Vaittinen P (1999) Parental age as a risk factor of childhood leukemia and brain cancer in offspring. *Epidemiol* **10**: 271–275

Hsieh C, Tzonou A and Trichopoulos D (1991) Birth order and breast cancer risk. *Cancer Causes Control* **2**: 95–98

Hsieh C, Thanos A, Mitropoulos D, Deliveliotis C, Mantzoros C and Trichopoulos D (1999) Risk factors for prostate cancer: a case-control study in Greece. *Int J Cancer* **80**: 699–703

Janerich DT, Thompson WD and Mineau GP (1994) Maternal pattern of reproduction and risk of breast cancer in daughters: results from the Utah population database. *J Natl Cancer Inst* **86**: 1634–1639

Juntunen K, Laara E and Kauppila A (1997) Grand grand multiparity and birth weight. *Obstet Gynecol* **90**: 495–499

Kaijser M, Granath F, Jacobsen G, Cnattingius S and Ekbom A (2000) Maternal pregnancy estriol levels in relation to anamnestic and fetal antropometric data. *Epidemiology* **11**: 315–319

Kaye SA, Robinson LL, Smithson WA, Gunderson P, King FL and Neglia JP (1991) Maternal reproductive history and birth characteristics in childhood acute lymphoblastic leukaemia. *Cancer* **68**: 1351–1355

Michels K, Trichopoulos D, Robins J, Rosner B, Manson J, Hunter D, Colditz G, Hankinson S, Speizer F and Willet W (1996) Birthweight as a risk factor for breast cancer. *Lancet* **348**: 1542–1546

Moller H and Skakkebaek N (1997) Testicular cancer and cryptorchidism in relation to prenatal factors: case-control study in Denmark. *Cancer Causes Control* **8**: 904–912

Potischman N and Trosi R (1999) In-utero and early life exposures in relation to risk of breast cancer. *Cancer Causes Control* **10**: 561

Sabroe S and Olsen J (1998) Perinatal correlates of specific histological types of testicular cancer in patients below 35 years of age: a case-cohort study based on midwives' records in Denmark. *Int J Cancer* **78**: 140–143

Sanderson M, Williams M, Malone K, Stanford J, Emanuel I, White E and Darling J (1996) Perinatal factors and risk of breast cancer. *Epidemiology* **7**: 34–37

Shaw G, Lavey R, Jackson R and Austin D (1984) Association of childhood leukemia with maternal age, birth order, and parternal occupation. *Am J Epidemiol* **119**: 788–795

Shu XO, Gao YT, Brinton LA and Al E (1988) A population-based case-control study of childhood leukemia in Shanghai. *Cancer* **62**: 635–644

Trichopoulos D (1990) Hypothesis: does breast cancer originate in utero? *Lancet* **335**: 939–940

Westergaard T, Andersen P, Pedersen J, Frisch M, Olsen J and Melbye M (1998) Testicular cancer risk and maternal parity: a population-based cohort study. *Br J Cancer* **77**: 1180–1185

© 2001 Cancer Research Campaign
